# The impact of text topic and assumed human vs. AI authorship on competence and quality assessment

**DOI:** 10.3389/frai.2024.1412710

**Published:** 2024-05-31

**Authors:** Sebastian Proksch, Julia Schühle, Elisabeth Streeb, Finn Weymann, Teresa Luther, Joachim Kimmerle

**Affiliations:** ^1^Department of Psychology, Eberhard Karls University Tuebingen, Tuebingen, Germany; ^2^Knowledge Construction Lab, Leibniz-Institut fuer Wissensmedien, Tuebingen, Germany

**Keywords:** large language models, ChatGPT, competence, quality assessment, morality, technology, algorithm aversion

## Abstract

**Background:**

While Large Language Models (LLMs) are considered positively with respect to technological progress and abilities, people are rather opposed to machines making moral decisions. But the circumstances under which algorithm aversion or algorithm appreciation are more likely to occur with respect to LLMs have not yet been sufficiently investigated. Therefore, the aim of this study was to investigate how texts with moral or technological topics, allegedly written either by a human author or by ChatGPT, are perceived.

**Methods:**

In a randomized controlled experiment, *n* = 164 participants read six texts, three of which had a moral and three a technological topic (predictor text topic). The alleged author of each text was randomly either labeled “ChatGPT” or “human author” (predictor authorship). We captured three dependent variables: assessment of author competence, assessment of content quality, and participants' intention to submit the text in a hypothetical university course (sharing intention). We hypothesized interaction effects, that is, we expected ChatGPT to score lower than alleged human authors for moral topics and higher than alleged human authors for technological topics and vice versa.

**Results:**

We only found a small interaction effect for perceived author competence, *p* = 0.004, *d* = 0.40, but not for the other dependent variables. However, ChatGPT was consistently devalued compared to alleged human authors across all dependent variables: there were main effects of authorship for assessment of the author competence, *p* < 0.001, *d* = 0.95; for assessment of content quality, *p* < 0.001, *d* = 0.39; as well as for sharing intention, *p* < 0.001, *d* = 0.57. There was also a small main effect of text topic on the assessment of text quality, *p* = 0.002, *d* = 0.35.

**Conclusion:**

These results are more in line with previous findings on algorithm aversion than with algorithm appreciation. We discuss the implications of these findings for the acceptance of the use of LLMs for text composition.

## 1 Introduction

The rise of ChatGPT and other Large Language Models (LLMs) has been referred to as a major step forward in Generative AI technology (Eke, 2023). It pushes “the boundaries of what is possible in natural language processing” (Kasneci et al., 2023, p. 2). LLMs are generators of text that appears like text written by humans. Such text is created by LLMs based on deep learning technology in response to people's prompts (Eke, 2023). In this context, the term creation means that the AI is able to generate content that is similar to the data with which it was trained, in this case texts. However, since this creation is essentially always prompted by human users, it can also be referred to as the co-construction of content (Cress and Kimmerle, 2023). LLMs can improve efficiency and workflow correcting syntactical and grammatical errors and provide short summaries of texts. According to Dwivedi et al. (2023), LLMs can even help with generating reasonable research hypotheses. ChatGPT can be used in almost any field, such as in the medical context (Cascella et al., 2023), in journalism (Pavlik, 2023), or in event planning (Keiper, 2023). However, LLMs also have their limitations (Deng and Lin, 2022; Salewski et al., 2024). For example, they might endanger academic integrity and the functionality of the review process of academic journals (Eke, 2023). Moreover, they often tend to reproduce biases, lack transparency, and might even reduce critical thinking by their users (Kasneci et al., 2023).

When users evaluate the performance of AI technology, a relevant factor is the magnitude of the consequences of its use. Bigman and Gray (2018), for example, found that people were averse to machines making moral decisions in several domains, including medicine, law, military, and autonomous driving. This aversion seemed to be quite robust and difficult to eliminate, even when machines were limited to a mere advisory role. Böhm et al. (2023) investigated whether an author label, that is, the fact that ChatGPT was labeled as the author of an article—as opposed to a human author—had any influence on how the texts were perceived. In their experiment, participants read short articles addressing societal challenges and evaluated the author's competence, the content quality of the text, and their intention to share the article with friends and family. Böhm et al. (2023) found a significant interaction effect of the author's identity and transparency on the author's competence. That is, whenever the author's identity (ChatGPT vs. human) was communicated to participants, they rated ChatGPT less competent than human authors. This effect vanished when the author's identity was not made transparent, indicating that people were not able to distinguish between texts written by people or AI. Lermann Henestrosa et al. (2023), however, did not find any differences between human-written and AI-written texts with respect to perceived credibility and trustworthiness of the texts. In a study by Köbis and Mossink (2021), participants had to distinguish whether various stories, news articles, and recipes were created by a human or an AI. This study found that people were unable to make this distinction, and they made their decisions at random.

The effectiveness of AI-generated content depends on how people evaluate the content and whether the advice is accepted at all. Current research is therefore focusing a lot on the question of whether and when people accept the help of algorithms. Contradictory statements can be found in the literature and opposing phenomena are shown (Hou and Jung, 2021). A large part of the research shows algorithm appreciation and refers to the tendency to give algorithmic content more weight than human content (Logg et al., 2019). The research on algorithm aversion, however, reveals the opposite effects (Dietvorst et al., 2015; Burton et al., 2020). Here, study results show that algorithmic content is increasingly rejected (Hou and Jung, 2021) or that people prefer human authorship over automated text generation (Lermann Henestrosa and Kimmerle, 2024).

In conclusion, there seems to be an ambivalence of how LLMs (and ChatGPT in particular) are perceived. On the one hand, LLMs are frequently associated with technological progress (e.g., Wei et al., 2022). Dwivedi et al. (2023), for example, point out that ChatGPT can successfully process more than eight programming languages. On the other hand, people are quite averse to machines making moral decisions (Bigman and Gray, 2018; Dietvorst and Bartels, 2022). In this study, we include the research results on algorithm aversion and appreciation. Therefore, the participants were presented with both texts on moral and on technological topics. The authorship label (ChatGPT vs. human) was randomly assigned. The texts had to be evaluated in terms of the author competence, the quality of the texts, and the intention to share the text in a hypothetical university course.

We expected interaction effects between the authorship label and text topic for each of the dependent variables.

H1: Interaction effect for author competence: ChatGPT's author competence will be evaluated better with a technological text topic, while a human's author competence will be evaluated better with a moral text topic.
H2: Interaction effect for content quality: content quality of a text that was labeled as written by ChatGPT will be evaluated better with a technological text topic, while the content quality of a text that was labeled as written by a human author will be evaluated better with a moral text topic.
H3: Interaction effect for sharing intention: sharing intention for a text that was labeled as written by ChatGPT will be stronger with a technological text topic, while sharing intention for a text that was labeled as written by a human author will be stronger with a moral text topic.

## 2 Materials and methods

The experiment that is presented here was preregistered on AsPredicted before the start of data collection (https://aspredicted.org/6ie9b.pdf).

### 2.1 Sample

We recruited *n* = 222 participants for this online experiment using university mailing lists. We terminated data collection on January 22, 2024. Participants were required to have an age of at least 18 years and have good knowledge of the German language. Exclusions of participants were performed according to the preregistered criteria: first, the data of participants were only used, if they gave their written informed consent and had completed the entire questionnaire. Second, we excluded participants who did not pass an attention check. For this, they had to pick the correct authors of all six texts (see procedure). A total of *n* = 58 participants were excluded from the analysis due to a failed attention check. This left us with *n* = 164 participants in the final dataset. Their mean age was *M* = 23.37 years (*SD* = 4.91); 130 were female, 30 male, and four non-binary persons.

### 2.2 Procedure

The experiment was composed in German and ran on www.soscisurvey.de. It started with a brief introductory part elaborating on anonymity, voluntariness, and participants' possibility to withdraw their own data without any consequences. Subsequently, participants were instructed to carefully read and evaluate the six texts they were going to be presented with sequentially.

On the next page, an authorship label appeared above the first text, which either indicated that the text was allegedly written by ChatGPT or by a human author (predictor authorship). For ChatGPT as author the label read: “The following text is from ChatGPT. ChatGPT (‘Chat Generative Pre-trained Transformer') is a text-based dialog system (‘chatbot') developed by the company OpenAI, which is based on machine learning.” For a human author the label stated: “The following text was written by a human author.” This label was randomly assigned to the texts—differently for each individual participant (with the restriction that each label appeared at least once per topic). Below the label, participants read the respective text that was either about a moral or technological topic (predictor topic). The order in which texts appeared was randomized across participants.

After reading each text, participants had to evaluate it regarding author competence, content quality, and their sharing intention. Subsequently, they had to answer the attention check question: “Which author wrote the text you have just read?” by clicking on “ChatGPT” or “human author” (the order of appearance was randomized). In a manipulation check question participants indicated which topic the text was about on a 7-point scale ranging from “moral” to “technological.” After passing through the six trials, participants were asked to report their age and gender. At the end of the experiment, participants could sign up for a drawing of seven vouchers for an online store (two vouchers worth 50 € each and five vouchers worth 20 € each). It took about 20 min to complete the entire experiment.

### 2.3 Material

Each text had a length of 200 words. The three moral texts were about *moral behavior, moral and globalization*, as well as *moral dilemmas*. The technological texts included the *technological revolution of the 21st century*, the role of *smartphones*, and the *future of technology*. The texts were composed using ChatGPT and the respective topic prompts. They were modified minimally to achieve a length of 200 words. To ensure that the quality of the presented texts was even among the texts, we conducted a pilot study with *n* = 34 participants. The results showed no meaningful differences in the quality of the texts. The mean scores of the texts were 3.85, 3.71, 3.80, 3.93, 4.01, and 3.78 respectively (rated on 5-point Likert-scales).

### 2.4 Measures

The measures of author competence and content quality were taken from Böhm et al. (2023). To measure participants' sharing intentions, we adapted a question from the same study. All items were measured on 7-point scales ranging from “1 = do not agree at all” to “7 = completely agree.”

*Author competence* was captured with three items: “The author is trustworthy,” “The author is knowledgeable of the subject,” and “The author is smart.”

*Content quality* was measured with five items: “The proposed solution described in the text is very concrete”, “The content of the text is very creative,” “The text is easy to understand,” “The text is well written,” and “The text is credible.”

To measure their *sharing intentions*, participants had to answer the prompt: “I would hand in this text as a student within a university course (in this scenario no legal consequences have to be considered).”

## 3 Results

With respect to the manipulation check item (7-point-scale from “1 = moral” to “7 = technological”), participants reported a mean of *M* = 1.71 (*SD* = 0.72) for moral texts and a mean of *M* = 6.25 (*SD* = 0.71) for technological texts, *t*_(163)_ = −50.48, *p* < 0.001. Hence, they have clearly recognized how the topics differed across texts.

To test the hypotheses, we deployed linear mixed-effects models with the two predictors *text topic* (“moral” coded as 0, “technological” as 1) and *authorship* (“ChatGPT” coded as 0, “human author” as 1). Linear mixed-effects models were fitted taking account the within-subjects design in the random effects. We tested separately for each of our three dependent variables. Mean scores were aggregated per condition per participant and per dependent variable. With Yi as the respective dependent variable, ε*i* as error terms, *i* = 1, 2, …, 164, and *j* = 1, 2, the corresponding model was conceptualized as follows: Y*i* = β0+β1 * *text topicij* + β2 * *authorshipij* + β3 * (*topicij* * *authorshipij*) + u0*j* + u1*j* * text *topicij* + u2*j* * authorship*ij* + ε*ij*.

### 3.1 Author competence

We found no main effect of text topic, β1 = 0.06, 95% CI (−0.08, 0.20), *SE* = 0.07, *t*_(206.23)_ = 0.86, *p* = 0.390. Alleged authorship, in contrast, had a strong impact on author competence, β2 = 0.78, 95% CI (0.62, 0.94), *SE* = 0.08, *t*_(413.85)_ = 9.69, *p* < 0.001, *d* = 0.95. That is, alleged human authors were granted significantly higher competence than ChatGPT. Finally, there was a significant interaction effect between text topic and alleged authorship, β3 = −0.28, 95% CI (−0.48, −0.09), *SE*=0.10, *t*_(206.23)_ = −2.89, *p* = 0.004, *d* = 0.40. However, this interaction effect was smaller than anticipated. Means and standard deviations of author competence by experimental condition can be found in Table 1.

**Table 1:** Means (*M*) and standard deviations (*SD*) of assessed author competence by experimental conditions.

	**Authorship: ChatGPT**	**Authorship: human author**
Moral text topic	*M* = 4.40, *SD* = 1.08	*M* = 5.18, *SD* = 0.86
Technological text topic	*M* = 4.46, *SD* = 1.09	*M* = 4.96, *SD* = 0.78

### 3.2 Content quality

Text topic had a small but significant main effect on content quality, β1 = 0.21, 95% CI (0.08, 0.34), *SE* = 0.07, *t*_(309.85)_ = 3.10, *p* = 0.002, *d* = 0.35, indicating that when authorship was held constant, the quality of the text was rated lower for moral than for technological topics. The predictor authorship influenced the rating of content quality, β2 = 0.30, 95% CI (0.16, 0.44), *SE* = 0.07, *t*_(468.93)_ = 4.18, *p* < 0.001, *d* = 0.39. Alleged human authors were associated with significantly higher content quality than ChatGPT. We did not find any significant interaction effects between text topic and authorship, β3 = −0.13, 95% CI (−0.32, 0.06), *SE* = 0.10, *t*_(309.85)_ = −1.34, *p* = 0.183. Means and standard deviations of content quality by experimental condition can be found in Table 2.

**Table 2:** Means (*M*) and standard deviations (*SD*) of assessed content quality by experimental conditions.

	**Authorship: ChatGPT**	**Authorship: human author**
Moral text topic	*M* = 4.56, *SD* = 0.92	*M* = 4.86, *SD* = 0.80
Technological text topic	*M* = 4.77, *SD* = 0.80	*M* = 4.94, *SD* = 0.72

### 3.3 Sharing intention

Text topic did not influence participants' sharing intention, β1 = −0.13, 95% CI (−0.39, 0.12), *SE* = 0.13, *t*_(303.33)_ = −1.02, *p* = 0.308. Authorship had a significant main effect on the sharing intention, β2 = 0.85, 95% CI (0.58, 1.12), *SE* = 0.14, *t*_(473.95)_ = 6.20, *p* < 0.001, *d* = 0.57. Participants were more willing to submit texts allegedly written by human authors than written by ChatGPT. We did not find a significant interaction effect between text topic and authorship, β3 = −0.26, CI (−0.63, 0.10), *SE* = 0.19, *t*_(303.33)_ = −1.41, *p* = 0.159. Means and standard deviations of sharing intentions by experimental condition can be found in Table 3. An overview of the fitted linear mixed-effects models is shown in Table 4.

**Table 3:** Means (*M*) and standard deviations (*SD*) of sharing intention by experimental conditions.

	**Authorship: ChatGPT**	**Authorship: human author**
Moral text topic	*M* = 4.07, *SD* = 1.64	*M* = 4.92, *SD* = 1.36
Technological text topic	*M* = 3.94, *SD* = 1.57	*M* = 4.52, *SD* = 1.49

**Table 4:** Overview of the fitted linear mixed-effects models.

**Predictors**	**Author competence**					**Content quality**					**Intention to share**				
	**Estimates**	***SE***	**CI**	***t***	***p*** **-value**	**Estimates**	***SE***	**CI**	***t***	***p*** **-value**	**Estimates**	***SE***	**CI**	***t***	***p*** **-value**
(Intercept)	4.40	0.08	4.25 to 4.55	58.44	< 0.001	4.56	0.06	4.44 to 4.68	73.07	< 0.001	4.07	0.12	3.84 to 4.30	34.90	< 0.001
Text topic	0.06	0.07	−0.08 to 0.20	0.86	0.390	0.21	0.07	0.08 to 0.34	3.10	0.002	−0.13	0.13	−0.39 to 0.12	−1.02	0.308
Authorship	0.78	0.08	0.62 to 0.94	9.69	< 0.001	0.30	0.07	0.16 to 0.44	4.18	< 0.001	0.85	0.14	0.58 to 1.12	6.20	< 0.001
Text topic × authorship	−0.28	0.10	−0.48 to −0.09	−2.89	0.004	−0.13	0.10	−0.32 to 0.06	−1.34	0.183	−0.26	0.19	−0.63 to 0.10	−1.41	0.159
**Random effects**															
σ^2^	0.40					0.38					1.41				
τ_00_	0.53 _id_					0.26 _id_					0.28 _id_				
τ_11_	0.00 _id.topic_					0.00 _id.topic_					0.00 _id.topic_				
ICC	0.27 _id.label_					0.07 _id.label_					0.26 _id.label_				
*N*	164 _id_					164 _id_					164 _id_				
Observations	656					656					656				
Marginal *R*^2^ /conditional *R*^2^	0.105/0.617					0.050/NA					0.097/NA				

## 4 Discussion

The experiment presented here studied the impact of text topic and alleged authorship on the assessment of author competence, content quality, and participants' intention to submit the text in a university course. We detected a small interaction effect only for perceived author competence, but not for the other dependent variables. That is, we did not find evidence for any of our interaction hypotheses. The predictor text topic seemed to have a small effect on content quality of the texts in that technological texts were ascribed a slightly higher quality than moral texts. An effect which remained robust throughout the experiment was the influence of alleged authorship. Participants rated human authors as more competent than ChatGPT, they found texts allegedly written by human authors to have a higher quality, and they were more willing to submit a text as their own term paper, if it was allegedly written by a human. It is important to bear in mind, however, that these were in fact always the same texts, only the label of the alleged authorship differed, and this alone was enough to produce very clear effects of authorship. Our data did not investigate how the participants' assessment would have changed if the texts had actually been written by ChatGPT vs. human authors.

Thus, our findings largely feed into the existing literature on algorithm aversion (Dzindolet et al., 2002; Dietvorst et al., 2015; Burton et al., 2020). Interestingly this effect was found even though the texts were all written by ChatGPT. Therefore, we suggest that the devaluation of ChatGPT's competence and text quality as well as the low intention to submit the text was not based on the fact that the ChatGPT texts were actually worse than those from a human author. This assessment rather was derived from people's thinking that ChatGPT's abilities were lower than those of humans regarding text production.

When it comes to LLMs helping to write articles, the benefits are obvious, as authors may apply various strategies (Luther et al., 2024) from collecting initial ideas, or providing content summaries to efficient proofreading, and translation into different languages. Nevertheless, Stanescu (2023) argues that the use of ChatGPT could foster a more rapid spread of fake news and disinformation, which might explain why participants were less willing to submit the alleged ChatGPT texts in our experiment.

To conclude, this study aimed at contributing to existing literature on perception and acceptance of AI tools such as LLMs. Though recent publications tended to find effects of algorithm appreciation, our findings based on participants' self-reports speak for an aversion to ChatGPT independently of the investigated topics.

## Data availability statement

The datasets presented in this study can be found in online repositories. The names of the repository/repositories and accession number(s) can be found below: https://osf.io/srvab/.

## Ethics statement

Ethical approval was not required for the studies involving humans, because this study was conducted as part of a course at the University of Tübingen and is therefore exempt from review by the local ethics committee. The studies were conducted in accordance with the local legislation and institutional requirements. The participants provided their written informed consent to participate in this study.

## Author contributions

SP: Conceptualization, Formal analysis, Methodology, Writing – original draft. JS: Conceptualization, Formal analysis, Methodology, Writing – original draft. ES: Conceptualization, Formal analysis, Methodology, Writing – original draft. FW: Conceptualization, Formal analysis, Methodology, Writing – original draft. TL: Conceptualization, Formal analysis, Methodology, Writing – review & editing. JK: Conceptualization, Funding acquisition, Methodology, Project administration, Writing – review & editing.
